# Development of a National Colorectal Cancer Screening Research Agenda: An Initiative of the Canadian Screening for Colorectal Cancer Research Network (CanSCCRN)

**DOI:** 10.3390/curroncol31120591

**Published:** 2024-12-18

**Authors:** Cynthia Kendell, Robin Urquhart, Akua Kyei, Steven J. Heitman, Jill Tinmouth

**Affiliations:** 1 Department of Medicine, Dalhousie University, Halifax, NS B3H 2Y9, Canada; 2 Nova Scotia Health, Halifax, NS B3S 0H6, Canada; robin.urquhart@nshealth.ca; 3 Department of Community Health and Epidemiology, Dalhousie University, Halifax, NS B3H 1V7, Canada; 4 Department of Medicine, Sunnybrook Health Sciences Centre, Toronto, ON M4N 3M5, Canadajill.tinmouth@sunnybrook.ca (J.T.); 5 Departments of Medicine and Community Health Sciences, Cumming School of Medicine, University of Calgary, Calgary, AB T2N 4Z6, Canada; sjheitma@ucalgary.ca; 6 Department of Medicine, University of Toronto, Toronto, ON M5S 3H2, Canada

**Keywords:** colorectal cancer, cancer screening, cancer surveillance

## Abstract

The Canadian Screening for Colorectal Cancer Research Network (CanSCCRN) recently set out to develop a national CRC screening research agenda and identify priority research areas. The specific objectives were to (1) identify evidence gaps relevant to CRC screening and the barriers and facilitators to evidence generation and uptake by CRC screening programs, (2) establish high-priority collaborative research ideas to inform best CRC screening practices, and (3) identify one to two research topics for grant development and submission within 12 to 18 months. Three focus groups were conducted with network members and relevant parties (*n* = 15) to identify evidence gaps, barriers, and facilitators to evidence generation and uptake. Three workshops were subsequently held to discuss focus group findings and develop an action plan for research. An electronic survey was used to prioritize the evidence gaps to be addressed. Overall, five categories of barriers and six categories of facilitators to evidence uptake and generation were identified, as well as 23 evidence gaps to be addressed. Screening participation, post-polypectomy surveillance, and screening age range were identified as research priority research areas. Adequate resourcing and infrastructure, as well as partnerships with knowledge end users, are integral to addressing these research areas and advancing CRC screening programs in Canada and beyond.

## 1. Introduction

### 1.1. Colorectal Cancer and Screening in Canada

Colorectal cancer (CRC) screening plays a critical role in the prevention and early diagnosis of CRC through the identification of precancerous polyps and asymptomatic invasive disease. Organized CRC screening, in particular—where an explicit screening policy is in place, as well as dedicated infrastructure, resources, and processes aimed at the screening of a specific target population [1,2]—is associated with increased screening uptake and improved outcomes, including reduced incidence and mortality [3,4,5,6]. Notably, previous modeling studies have suggested that increasing CRC screening uptake has a greater impact on the reduction of CRC mortality than advances in treatment [7], further underscoring the value of organized screening.

In Canada, the national incidence rates of CRC have been declining over the last several decades, with the most notable decline occurring since 2014 (−4.0% in men and −3.1% in women per year) [8]. This has been largely attributed to increased screening uptake resulting from the implementation of organized CRC screening programs in most Canadian jurisdictions. Currently, nine provinces (all except Quebec) and one territory (Yukon) have organized CRC screening programs in place [9]. These programs all employ fecal-based testing but vary in other respects, including the type and brand of test used, testing cutoffs, and approaches to follow up for abnormal results. There are also substantial variations in screening participation rates across the country. As highlighted in a recent report by the Canadian Partnership Against Cancer (CPAC), screening uptake among eligible individuals falls below the national target of 60% across all provinces and territories, ranging from 20.4% in NL to 50.6% in AB [9]. Overall, low screening participation rates, combined with the sobering fact that CRC remains the third most diagnosed cancer and the second leading cause of cancer-related death in Canada [8], emphasizes the need for ongoing efforts to optimize CRC screening to improve patient outcomes.

### 1.2. Advancing the Colorectal Cancer Screening Research Agenda

In 2019, researchers and clinicians with expertise in CRC screening came together with CRC screening program personnel to form the Canadian Screening for Colorectal Cancer Research Network (CanSCCRN). CanSCCRN was born out of the recognition that research is integral to optimizing the delivery of organized CRC screening programs in Canada and beyond and ensuring the benefits of organized CRC screening are fully realized. By bringing together decision-makers and expert researchers in the field, we aim to conduct collaborative, innovative, and methodologically rigorous research that is highly relevant to organized screening programs and to facilitate the uptake of research findings, thereby optimizing the benefits of CRC screening for all Canadians. As a pan-Canadian network, we are well-positioned to contribute to this research by leveraging provincial/territorial programs as ‘learning health systems’ [10]. The widespread implementation of organized screening programs across the country and the differences in program elements and implementation contexts are well suited to the conduct of “natural experiments” to facilitate the evaluation of current practices, along with the development and testing of targeted strategies for improvement.

Currently, CanSCRRN has 40 members from across Canada, including researchers, clinicians, provincial/territorial CRC screening program staff, and administrators—all of whom have a vested interest in advancing CRC screening through research. With representation from most Canadian provinces and territories, members meet regularly via virtual videoconferencing software (every 3 to 5 months) and in person when opportunities arise. The Network does not receive funding from provincial or federal governments and is sustained through grant funding for specific projects. In addition to providing a forum for screening programs to discuss challenges being faced in the CRC screening space and how to address these, the Network has collaborated on several key initiatives relevant to CRC screening research, including measuring the performance of Canadian provincial/territorial screening programs using international metrics [11] and approaches to optimizing the quality of colonoscopy for Canadian provinces/territories (in progress).

To guide CanSCRRN’s program of research moving forward and to ensure the research being undertaken by the network aligns with the needs of knowledge users, a team of network members (led by JT, RU, and SJH) obtained funding from the Canadian Institutes of Health Research (CIHR) to carry out activities to foster collaboration between Canadian provincial/territorial organized CRC programs, researchers with expertise in CRC screening, and other interested parties, and to gather information that would inform the network’s research agenda. Specifically, the team set out to:

(1) Identify evidence gaps relevant to CRC screening and the barriers and facilitators to the generation and uptake of research evidence by CRC screening programs;
(2) Establish high-priority collaborative research ideas to inform best CRC screening practices in Canada and beyond, and
(3) Identify one to two collaborative research topics for grant development and submission within the next 12 to 18 months.

The purpose of this paper is to describe the work carried out to address these objectives.

## 2. Methods

A phased approach was employed, involving focus groups followed by a series of workshops and the administration of an online survey. The focus groups were approved by the Sunnybrook Health Sciences Research Ethics Board, while the latter did not require REB approval.

### 2.1. Focus Groups

Three focus groups were undertaken in March 2023 to identify evidence gaps and the barriers and facilitators to the generation and uptake of evidence by CRC screening programs. Participants (*n* = 15) were identified purposively by the research team and included individuals in various roles (clinicians, researchers, screening program personnel, etc.) with expertise relevant to CRC screening research and program organization. Invitees included CanSCCRN members, as well as individuals who were not part of the Network (e.g., from relevant organizations such as CPAC, Colorectal Cancer Canada, and the Canadian Association of Gastroenterology). Participants included individuals from seven provinces, approximately half from Ontario (47%) and the remainder from British Columbia, Alberta, Manitoba, Quebec, Nova Scotia, and Newfoundland. These individuals were invited to participate via email, and written informed consent was obtained prior to focus group participation.

Focus groups were held virtually and conducted by a team member with extensive qualitative research experience. A separate focus group was held for each of the following groups: researchers (*n* = 7); CRC screening program planners/administrators (*n* = 5); and other interested parties (e.g., provincial primary care leads; *n* = 3). The focus group guide [Supplementary File SA] was developed based on practical guidance from Patton [12] with questions informed by the Consolidated Framework for Implementation Research (CFIR) [13]. A semi-structured approach was used, allowing the order and wording of questions to be adapted based on the flow of conversation and allowing the facilitator to “probe” as needed (i.e., to ask additional follow up questions to obtain additional information or clarifications). Focus groups were audio-recorded and transcribed verbatim to facilitate analysis. Data analysis was performed by the research coordinator with oversight and additional input from senior team members to ensure methodological rigor and consistency. During the coding process, a subset of transcripts was independently reviewed by two team members to assess agreement on the application of codes and enhance reliability. Differences in coding were resolved through discussion until a consensus was reached. A general thematic analysis was undertaken, employing an approach consistent with that of Braun and Clark [14]. Transcripts were iteratively reviewed to assess the depth and breadth of the information. During the initial coding phase, significant passages of text were highlighted, and codes were assigned based on the content. These codes represented basic units of meaning, capturing key ideas, concepts, and issues raised by the participants. The codes were subsequently examined to identify patterns and similarities. Related codes were grouped into subthemes, which were further analyzed and combined into overarching themes that represented broader concepts encapsulating the essence of the data. The themes were then reviewed (i.e., for consistency, coherence, clarity, and precision), revised, named, and summarized.

### 2.2. Workshops

**Figure 1:**
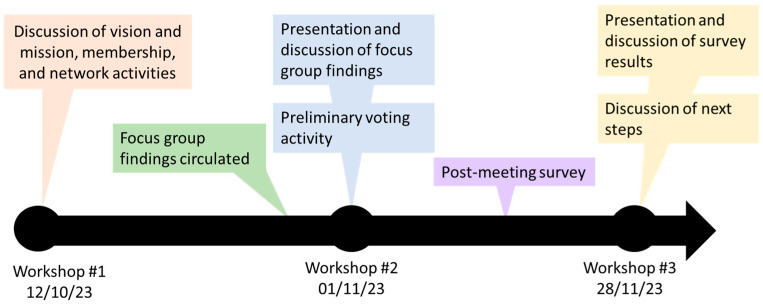
Timeline of workshops and related activities.

In Fall 2023, a series of virtual workshops were held with CanSCCRN members to develop a national research agenda for CRC screening. Building on focus group findings, the workshops aimed to guide attendees in ultimately identifying one to two high-priority research topics on which team members could collaborate and develop proposals for submission to national funding competitions. All CanSCCRN members (*n* = 39) were invited by email to attend and were encouraged to share the invitation with other interested parties with relevant expertise within their networks. The non-CanSCCRN members who participated in the focus groups were also invited to attend the workshops. In total, three workshops were held over a two-month timeframe (October to November). These were guided by a team member with prior facilitation experience as well as prior CRC research experience. Attendance varied across the workshops (workshop 1, *n* = 18; workshop 2, *n* = 20; workshop 3, *n* = 16), with a total of 32 individuals participating in at least one workshop. More than half of these individuals attended more than one workshop (56%). For each workshop, attendees included individuals in various roles (e.g., clinicians [primary care providers, gastroenterologists, oncologists], researchers [health services, biomedical], screening program planners/administrators, etc.) and represented most Canadian jurisdictions. A representative from each jurisdiction attended at least one workshop, with the exception of New Brunswick, Prince Edward Island, the Yukon, and Nunavut. In addition to the three workshops, a postworkshop survey was administered after workshop #2. Participants were also invited to share feedback and reflections with the team via email at any time. These activities are described below, and a timeline is provided in Figure 1.

- *Workshop 1*—Designed to develop rapport between network members and establish agreement on the purpose of the network and how it would operate, this workshop was organized around three key discussion topics: (1) network vision and mission, (2) membership expansion and diversification, and (3) general network activities (i.e., communication strategy, meeting frequency, etc.).
- *Workshop 2*—Focus group findings were presented to attendees (who had received a summary of findings several days prior) and discussed. Attendees were asked to share their perspectives on the findings, including whether they were consistent with their own experiences/perspectives and whether there were specific evidence gaps identified during focus groups that the network was particularly well positioned or not well positioned to address. Finally, a preliminary online voting activity was undertaken (using the Whiteboard feature within Zoom for Healthcare). The evidence gaps identified during the focus groups were listed, and participants were asked to identify which evidence gap (framed as potential research topics) they would be most interested in studying and to add any additional topics that were not reflected in the original list.
- *Postworkshop survey*—Based on the focus group findings, the second workshop discussion, and the preliminary voting exercise, an electronic survey was developed and administered to CanSCCRN members, focus group participants, and other meeting attendees (*n* = 52) via email approximately one week after the second workshop. In the survey, the evidence gaps identified during the focus groups, with minor modifications based on the discussion that occurred during the second workshop, were listed as potential research topics, and participants were asked to score each topic based on importance (1 = least important, 10 = most important). The survey remained open for two weeks. Mean scores were calculated and used to rank topics from highest to lowest importance. A summary of survey results was circulated to CANSCCRN members and workshop attendees in advance of the third workshop.
- *Workshop 3*—Survey results were presented and discussed. With a focus on the top-ranked topics (mean score > 8), the discussion focused on (1) identifying one to two topics for submission to a national funding competition, (2) identifying team members and project leads, and (3) determining next steps. An overview of the sequence of workshops and related activities, including timelines, is depicted in Figure 1.

## 3. Main Outcomes

### 3.1. CanSCCRN Vision and Mission Statements

**Figure 2:**
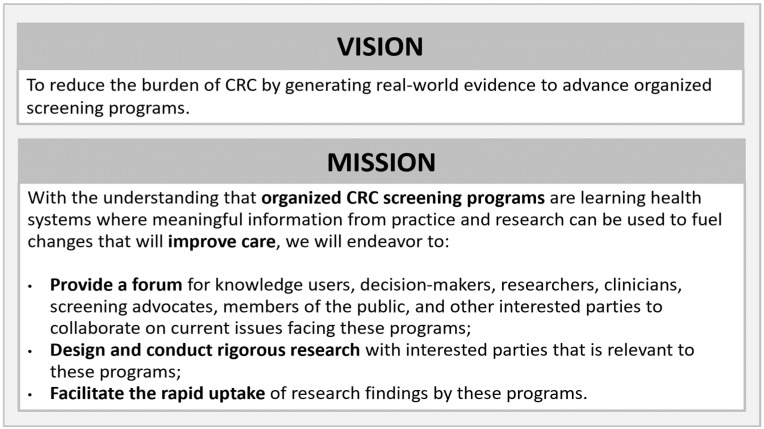
CanSCCRN vision and mission statements.

Draft versions of the vision and mission statement were presented at the outset of the first workshop and subsequently revised based on input from attendees during the workshop and received via email. These were presented during the third workshop and approved by those in attendance. These statements, intended to guide the work of the Network, are provided in Figure 2.

### 3.2. Focus Group Findings

**Figure 3:**
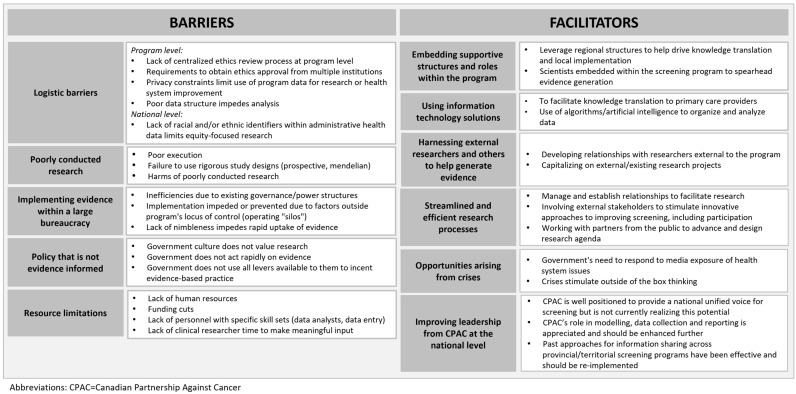
Barriers and facilitators to evidence generation and uptake by provincial screening programs.

A multitude of barriers and facilitators to evidence generation and uptake were identified by focus group participants, which were subsequently organized into five categories of barriers and six categories of facilitators. These are summarized in Figure 3. Of the six facilitators that were identified, improving leadership from CPAC was discussed as a potential facilitator. Participants voiced their opinions that as the steward for Canada’s cancer control strategy, CPAC had an important role in facilitating CRC screening research and the uptake of this research at the national level. At the same time, participant responses indicated the belief that CPAC was not currently acting in their full capacity and should be doing more to facilitate evidence generation and uptake relevant to CRC screening.

### 3.3. Ranked Research Topics

Participants scored a total of 23 research topics in the postworkshop survey. This included the evidence gaps identified during focus groups, plus one additional research topic identified during the second workshop (i.e., “Examining differences in colorectal cancer screening programs across provinces, in terms of organization and delivery, participation, and outcomes”). Responses were received from 34 individuals for a 65% response rate. Respondents represented 11 of 13 Canadian jurisdictions, with the greatest proportion of responses from Ontario (38.2%), followed by Nova Scotia (11.8%), and Quebec and Alberta (8.8% for each). The research topics included in the survey, along with the mean score and resulting rank, are provided in Table 1. Overall, topics were scored quite highly, with mean scores ranging between 5.91 and 8.49. Of the topics that scored highest for importance (i.e., those with a mean score > 8), the majority related to increasing participation in screening (i.e., five out of six topics). Several of these topics were interrelated; for example, determining how to identify under-screened populations is intertwined with developing and implementing culturally specific strategies to improve the participation of under-screened populations. Notably, while ranks were assigned based on mean score, the margin of difference between many of the topics listed was quite small.

## 4. Discussion

### 4.1. Identifying Priority Areas for Research

The findings from the focus groups revealed that the individuals responsible for CRC screening program planning and delivery in Canada have wide-ranging evidence needs. The mean scores calculated based on the postworkshop survey were quite high, with most potential research topics scoring > 7. This reflects the high relevance of these topics as identified by the workshop participants themselves. While many of the research topics identified in this paper would warrant attention, the survey results and subsequent discussions highlighted three key areas to address: screening participation, post-polypectomy surveillance, and screening age range.

The specific topics related to increasing screening participation that were considered the highest priority included developing and implementing strategies to improve screening uptake (broadly and in under-screened populations), identifying barriers and facilitators to screening for under-screened populations, and determining how to identify under-screened populations. The prioritization of topics related to increasing screening uptake was not unexpected, given the current low screening rates across the country [9]. The question of how to improve screening uptake remains a challenge for many jurisdictions beyond Canada. A 2019 systematic review examining the effectiveness of interventions to improve participation in mail-out CRC screening programs [15] identified four categories of interventions for effectively increasing screening uptake: advance notification, general practitioner endorsement, telephone contact, and the simplification of testing procedures. However, these interventions only produced a moderate increase in uptake. The authors noted the need for additional research to understand the mechanisms by which these interventions were successful and how they might be applied to different settings, as well as the need for novel interventions (or combinations thereof) to achieve desired screening levels. The need for novel approaches is certainly relevant in the Canadian context, given that many jurisdictions have already implemented the aforementioned evidence-based interventions to improve screening uptake [9] yet continue to experience suboptimal participation rates.

Addressing screening uptake in under-screened populations was considered particularly important by workshop participants. Given variations in screening participation rates across various groups (e.g., by income and across immigrant groups and geographic settings) [9], targeted interventions are needed that address the specific barriers to screening faced by those groups with low uptake. However, data related to race, ethnicity, and other social determinants of health that may impact screening participation are not routinely collected by provincial screening programs or healthcare systems in Canada, limiting our understanding of who is not being screened and ways to improve uptake in these groups. Even if these data were available, determining how to accurately define specific groups (race, ethnicity, or other) is not straightforward, as the group to which an individual is assigned may not align with the individual’s identity [16]. These data gaps limit the extent to which disparities and inequities across subgroups can be identified and hinder the ability to develop, implement, and evaluate evidence-based interventions aimed at improving screening uptake among those who may experience greater barriers. Related to this, CPAC has highlighted a lack of research on screening uptake and barriers to screening among specific subgroups (i.e., including individuals with disabilities, chronic disease, and mental health conditions, as well as individuals who are transient, experiencing homelessness, and LGBTQ2S+) that may be considered for future inquiry [9].

Workshop participants identified several evidence needs best unified under the broader theme of post-polypectomy surveillance, including evidence related to follow up for high-risk adenomas, the use of FIT testing for low-risk adenomas, and surveillance of serrated sessile lesions. While there are multiple guidelines for post-polypectomy surveillance that encompass adenomas and sessile serrated lesions (e.g., [17,18,19]), these have important limitations. Specifically, current classifications of adenomas (i.e., “high” or “low” risk) are not supported by strong evidence [20], leading to variations in how adenomas are classified and subsequently managed. For example, guidelines in Ontario and Alberta use different definitions for what is considered “high” and “low” risk [17,18], while the United Kingdom employs a third category of adenomas that are considered “intermediate risk” [16]. The recommended approach to the management of low-risk adenomas also varies by guideline, with some jurisdictions recommending colonoscopy [21] and others, including many Canadian provinces, recommending follow up with FIT (e.g., Ontario [17], Alberta [18], British Columbia [22]). The latter recommendation results from the observation that persons diagnosed with low-risk adenomas are at similar or lower risk of dying from CRC than those who are at average risk in the general population [23], and therefore, the same test (i.e., FIT) should be used. That said, there are limited data on the use of FIT in those with low-risk adenomas; therefore, studies on its effectiveness and implementation (e.g., timing after the index colonoscopy, intervals, threshold, etc.) are needed. Finally, there is also limited evidence to support specific recommendations about the management of sessile serrated lesions beyond the initial colonoscopy, resulting in cross-jurisdictional variations in the timing and frequency of subsequent procedures.

The prioritization of evidence related to the screening age range is driven by the rapidly increasing rates of early onset CRC (eoCRC) in Canadians under the age of 50 years [24,25], calling into question current provincial guidelines [9], which recommends screening for average-risk individuals over the age of 50 [26]. In the United States, the observed increase in eoCRC has resulted in updated guidelines recommending that screening start at 45 years of age [27]. The need for evidence to inform decision-making regarding whether to implement similar changes in Canada has also been noted by Brenner et al. [28], specifically regarding the characteristics of eoCRC (histology, topography, stage at diagnosis, and mortality), the cost-effectiveness of an earlier screening age, eoCRC risk profiles, and the effectiveness of current and novel tests of biomarkers in the 40 to 49 age group.

### 4.2. Mitigating Barriers to Evidence Generation and Uptake

The focus groups identified various barriers to evidence generation and uptake, including logistical challenges, poor quality research, institutional bureaucracy, government culture, and resource limitations. These barriers are similar to those that have been previously identified in Canada [29] and other jurisdictions for health research more broadly [30,31,32].

Several of these barriers may be addressed through the collaborative approach to research being employed by CanSCCRN. While there are many evidence gaps relevant to CRC screening, provincial/territorial screening programs themselves typically have limited capacity to undertake research to address these—or to navigate the related logistical and bureaucratic challenges—due to resource limitations, including a lack of funding, limited human resources to acquire funding and conduct the research, and time constraints faced by clinicians and other program personnel. CanSCCRN’s strength lies in uniting clinicians, researchers, and CRC screening program staff and administrators from across the country, resulting in a pooling of skills and expertise, which can be leveraged to obtain research funding, navigate logistical challenges (i.e., obtaining ethics, privacy constraints), and conduct high-quality research. Moreover, this collaboration ensures that the research being undertaken is relevant to the knowledge end users, thereby increasing the likelihood that findings will be integrated into program delivery at the provincial/territorial level.

Other barriers to evidence generation and uptake may be more challenging to address, including those related to data capture and data infrastructure, institutional bureaucracy, and government culture. Data-related barriers to research in Canada are well documented [33,34,35,36], and in recent years, there have been various provincial and national initiatives aimed at improving data collection and optimizing the utility and accessibility of these data for research [37,38,39]. As these initiatives progress and the data landscape evolves, new opportunities may arise for CRC screening research that CanSCCRN will be well-prepared to pursue. In the short term, data limitations may be addressed through novel data linkages and statistical approaches (e.g., Bayesian statistics [40]), as well as the use of primary data collection to supplement existing data sources.

Institutional bureaucracy and government culture also hinder the uptake of research evidence. Screening programs have limited decision-making authorities and are subject to many layers of institutional oversight, which can lead to challenges in implementing changes to program delivery. In many provinces, this is compounded by what participants perceive to be a government culture that undervalues research, as reflected in a lack of incentives to encourage evidence-based practice, and decision and policy-making. Potential solutions to these issues are much less clear. Culture, of which policies and processes are a reflection [41], is closely tied to leadership (i.e., the attitudes and values of those ‘in charge’) [42], underscoring the importance of getting “buy-in” from relevant health system leaders regarding the value of CRC screening, and research to inform screening. As such, bureaucratic and cultural barriers may be mitigated over time as the network expands to engage a broader range of interested parties, builds relationships with health system decision-makers, and establishes its role as a leader in CRC screening research.

### 4.3. Next Steps for CanSCCRN

The next step for CanSCCRN is to identify one to two specific research projects based on the broad priority areas identified to serve as the basis for grant development and submission to a national funding competition within the next 12 to 18 months. While there is a clear interest in the broad topics discussed above (screening participation, post-polypectomy surveillance, and screening age range), the specific research questions for which grant funding will be pursued have yet to be determined. These will be selected based on several considerations, including interest from CanSCCRN members, alignment with provincial/territorial priorities, and available funding opportunities. To avoid the duplication of efforts, ongoing work by other national organizations, such as CPAC and the Canadian Association of Gastroenterology, will also be considered.

Importantly, sustaining the work of CanSCCRN will require resources beyond project-specific funding. Specifically, there is a need for personnel and funds to support the core infrastructure of the organization. Key functions initially would include communication (including email and website), organizing membership, meetings, and support for grant applications related to the research agenda. In the future, as the organization expands, analytic and statistical support will be required, especially for projects that link large datasets from multiple provinces and territories and those where data harmonization is required.

A limitation of the work presented in this manuscript is the lack of representation from certain groups in the work described in this manuscript. While every effort was made to secure representation from all Canadian jurisdictions in each step of the priority setting process, this did not occur (e.g., New Brunswick, Prince Edward Island, the Yukon, and Nunavut were not represented at the workshops). As such, the research priorities that were identified may not align with the evidence needs in all jurisdictions. Additionally, the perspectives of patients and public partners were largely absent from the work described in this manuscript due to their lack of engagement in the Network. This lack of engagement, particularly with historically marginalized communities and equity-seeking groups (e.g., racialized groups; First Nations, Inuit, and Métis; newcomers; low-income, etc.) is such that the needs of these communities/groups may not be reflected in the current research agenda. Moving forward, efforts to expand network membership will focus on recruiting a broader range of members, including patient and public partners from diverse backgrounds, to ensure their unique needs and perspectives are represented in and addressed by the research undertaken by the Network.

## 5. Conclusions

Research is integral to optimizing CRC screening programs, reducing CRC incidence, and improving patient outcomes through early diagnosis and intervention. This paper describes efforts undertaken by CanSCCRN members and other interested parties to develop a national research agenda to inform CRC screening using a systematic and collaborative approach, incorporating direct input from knowledge end users at each step to meet their evidence needs. Through the identification of evidence gaps and ranking based on perceived importance, we have identified three broad priority areas for research that represent the pragmatic issues faced by CRC screening programs in Canada. The engagement of various groups in this work was not only integral to identifying these priority areas but also to building and strengthening the relationships required for successful collaboration. Moving forward, we will continue to establish new relationships and partnerships as well as build the necessary infrastructure to design and conduct rigorous research to advance CRC screening programs in Canada and beyond.

## Figures and Tables

**Table 1:** Ranking of research topics based on mean score.

Rank #	Research Topic	Mean Score
1	Developing and/or implementing culturally specific/targeted strategies to improve the participation of under-screened populations	8.49
2	Developing and/or implementing evidence-based targeted approaches to improve overall screening participation	8.39
3	Identifying barriers and facilitators to screening for under-screened populations (e.g., language, distrust, personal or historical ways)	8.21
4	Developing strategies to improve FIT uptake and return (e.g., direct FIT mailing and reminders)	8.15
5	Investigating the etiology of early onset colorectal cancer and implications for screening (e.g., whether to commence screening at an earlier age)	8.12
6	Determining how to identify under-screened populations to support the evaluation of strategies aimed at improving screening participation	8.06
7	Refining follow up protocols for high-risk adenomas (e.g., type and frequency of testing, revisiting the definitions of high-risk adenoma)	7.97
8	Defining optimal age range for screening	7.76
9	Generating evidence to support the surveillance of sessile serrated lesions	7.73
10	Generating evidence to support and refine the use of FIT after low-risk adenomas (e.g., FIT effectiveness, how long after colonoscopy)	7.64
10	Exploring strategies to support knowledge translation for post-polypectomy surveillance	7.64
11	Determining when to stop surveillance (e.g., what age, severity of most recent precancerous finding)	7.61
12	Identifying “best practices” for screening program implementation (e.g., exploring strategies to make the system easier for patients and providers)	7.57
13	Exploring alternative algorithms for FIT (e.g., adjusting intervals based on prior test results)	7.55
14	Exploring or evaluating new tests to screen for colorectal cancer (e.g., blood-based testing, multicancer tests, tests to supplement FIT)	7.33
15	Developing/and or implementing strategies to address the colonoscopy backlog (e.g., using nonphysician endoscopists, evidence-based prioritization schemes)	7.18
16	Exploring physician behavior (e.g., what motivates physicians to change behavior, how to incentivize behavior change)	7.06
17	Describing the cost-benefit ratio of increasing screening participation past current participation rates	6.36
18	Examining how to offer informed participation in the context of our current approach to programmatic screening	6.82
19	Exploring the use of artificial intelligence for colonoscopy	6.73
20	Examining differences in colorectal cancer screening programs across provinces, in terms of organization and delivery, participation, and outcomes	6.55
20	Refining patient comfort/satisfaction metrics specific to colonoscopy	6.55
21	Exploring the use of FIT in symptomatic individuals	5.91

## Data Availability

Focus group data are not publicly available to maintain the privacy of research participants. Additional data collected as part of the work outlined in this manuscript may be available upon reasonable request to the authors.

## Supplementary Materials

The following supporting information can be downloaded at: https://www.mdpi.com/article/10.3390/curroncol31120591/s1. Supplementary File SA. Focus group guide.
