# Supplementary material for: Development of a National Colorectal Cancer Screening Research Agenda: An Initiative of the Canadian Screening for Colorectal Cancer Research Network (CanSCCRN)
Source: Curr Oncol. 2024 Dec 18;31(12):8010–22. doi: 10.3390/curroncol31120591 (PMC11675037; doi:10.3390/curroncol31120591)
Supplement: Supplementary file 1 [file curroncol-31-00591-s001.zip › curroncol-3329247-supplementary.pdf]

**Supplementary file SA. Focus group guide**

The questions below were adapted based on the specific focus group participants.

| |
|---|
| ***Evidence needs (program planners/decision-makers only)***<br>We are interested in learning about your needs in terms of what information and data you require to effectively run your programs and to make good decisions. This will allow us to guide the research community to develop and tailor their research questions in ways that are relevant to screening programs |
| Tell me about your **familiarity** and **comfort** with using research evidence in program planning and refinement.<br>*Probes:*<br>• To what extent do you feel research evidence is needed to run your program?<br>• How do you identify relevant research evidence?<br>• How do you go about using this evidence?<br>• What other types of evidence do you use (e.g., quality improvement initiatives, information<br>• from other programs, etc)? |
| What are some **key gaps** in the evidence base that create challenges to running your program?<br>*Probes:*<br>• FIT cut-offs and impacts on detection rates<br>• Effectiveness of different invitation strategies<br>• Reaching underserved populations<br>• Health services gaps/inefficiencies |
| Can you describe any quality improvement initiatives that your program is currently undertaking/has recently undertaken? |
| Can you describe the approach to equity, diversity and inclusion (EDI) in your program?<br>*Probes:*<br>• Are there any specific EDI initiatives that you are aware of? |
| ***Barriers and facilitators to generating evidence (all participants)***<br>We would now like to understand specific barriers and facilitators to generating evidence for CRC screening programs. Barriers and facilitators may occur at different levels of the health care system, from individual physicians to system-level governance and regulation. This perspective is important and will allow us to try and build capacity in CRC screening research by targeting key barriers and facilitators. |
| What **barriers** exist in your program and province to generating research evidence for CRC screening?<br>*Probes:*<br>• Innovation: relative advantage that research provides (or not) over existing or other sources of information, degree to which research can be adapted to meet local needs, perceived difficulty of research, costs of research<br>• Inner setting: relative priority of research compared to other initiatives, degree of fit with departmental/organizational interests and priorities, perceived (dis)incentives to research, available resources (e.g., funds, available researchers and skilled staff/analysts), degree of commitment (from senior management), organizational culture, ease of data access and sharing<br>• Outer setting: policy and regulations, research governance (e.g., privacy legislation, REB approvals), external recommendations, resource issues, pressing/competing priorities<br>• Process: relationship to/trust in researchers, engagement of appropriate individuals (e.g., credible researchers and other stakeholders) |

| |
|---|
| What **facilitators** exist in your program and province to generating research evidence for CRC screening?<br>*Probes:*<br>• Innovation: relative advantage that research provides (or not) over existing or other sources of information, degree to which research can be adapted to meet local needs, perceived difficulty of research, costs of research<br>• Inner setting: relative priority of research compared to other initiatives, degree of fit with departmental/organizational interests and priorities, perceived (dis)incentives to research, available resources (e.g., funds, available researchers and skilled staff/analysts), degree of commitment (from senior management), organizational culture, ease of data access and sharing<br>• Outer setting: policy and regulations, research governance (e.g., privacy legislation, REB approvals), external recommendations, resource issues, pressing/competing priorities<br>• Process: relationship to/trust in researchers, engagement of appropriate individuals<br>• (e.g., credible researchers and other stakeholders) |
| Out of all of the things you have discussed in this focus group, what do you think are the **critical** barriers and facilitators to generating evidence? |
| ***Summing up*** |
| Is there anything else related to evidence needs or gaps, or barriers and facilitators to generating evidence, that we haven't mentioned that you believe should be brought up today? |
